# Survival of patients with ruptured gastrointestinal stromal tumour treated with adjuvant imatinib in a randomised trial

**DOI:** 10.1038/s41416-024-02738-z

**Published:** 2024-06-11

**Authors:** Heikki Joensuu, Annette Reichardt, Mikael Eriksson, Peter Hohenberger, Kjetil Boye, Silke Cameron, Lars H. Lindner, Philipp J. Jost, Sebastian Bauer, Jochen Schütte, Stefan Lindskog, Raija Kallio, Panu M. Jaakkola, Dorota Goplen, Eva Wardelmann, Peter Reichardt

**Affiliations:** 1 Department of Oncology, Helsinki University Hospital and University of Helsinki, Helsinki, Finland; 2 Helios Klinikum Berlin-Buch, and Berlin Medical School, Berlin, Germany; 3 Department of Oncology, Skåne University Hospital and Lund University, Lund, Sweden; 4 Division of Surgical Oncology & Thoracic Surgery, Mannheim University Medical Center, Mannheim, Germany; 5 Department of Oncology, The Norwegian Radium Hospital, Oslo University Hospital, Oslo, Norway; 6 Department of Gastroenterology and Gastrointestinal Oncology, University Medicine Göttingen, Göttingen, Germany; 7 Department of Medicine III, University Hospital, LMU Munich, Munich, Germany; 8 Medical Department III, Klinikum rechts der Isar, Technical University of Munich, Munich, Germany; 9 Division of Clinical Oncology, Department of Internal Medicine, Medical University of Graz, Graz, Austria; 10 Department of Medical Oncology and Sarcoma Center, West German Cancer Center, University Duisburg-Essen, Medical School, Essen, Germany; 11 DKTK partner site Essen, German Cancer Consortium (DKTK), Heidelberg, Germany; 12 Schwerpunktpraxis Oncology/ Hematology, Düsseldorf, Germany; 13 Universitätsklinikum Essen Innere Klinik Essen, Essen, Germany; 14 Department of Surgery, Institute of Clinical Sciences, Sahlgrenska Academy, University of Gothenburg, Gothenburg, Sweden; 15 Department of Surgery, Halland Hospital, Varberg, Sweden; 16 Department of Oncology and Radiotherapy, Oulu University Hospital, Oulu, Finland; 17 Department of Oncology, Turku University Hospital and University of Turku, Turku, Finland; 18 Haukeland University Hospital, Bergen, Norway; 19 Gerhard-Domagk-Institute of Pathology, University of Münster, Münster, Germany

**Keywords:** Sarcoma, Targeted therapies, Molecular medicine, Molecularly targeted therapy

## Abstract

**Background:**

Patients with ruptured gastrointestinal stromal tumour (GIST) have poor prognosis. Little information is available about how adjuvant imatinib influences survival.

**Methods:**

We explored recurrence-free survival (RFS) and overall survival (OS) of patients with ruptured GIST who participated in a randomised trial (SSG XVIII/AIO), where 400 patients with high-risk GIST were allocated to adjuvant imatinib for either 1 year or 3 years after surgery. Of the 358 patients with confirmed localised GIST, 73 (20%) had rupture reported. The ruptures were classified retrospectively using the Oslo criteria.

**Results:**

Most ruptures were major, four reported ruptures were reclassified unruptured. The 69 patients with rupture had inferior RFS and OS compared with 289 patients with unruptured GIST (10-year RFS 21% vs. 55%, OS 59% vs. 78%, respectively). Three-year adjuvant imatinib did not significantly improve RFS or OS of the patients with rupture compared with 1-year treatment, but in the largest mutational subset with *KIT* exon 11 deletion/indel mutation OS was higher in the 3-year group than in the 1-year group (10-year OS 94% vs. 54%).

**Conclusions:**

About one-fifth of ruptured GISTs treated with adjuvant imatinib did not recur during the first decade of follow-up. Relatively high OS rates were achieved despite rupture.

**Clinical Trial Registration:**

NCT00116935.

## Background

Gastrointestinal stromal tumour (GIST) is one of the most common types of soft-tissue sarcoma and the most common sarcoma of the gastrointestinal tract [1]. GIST may arise anywhere along the gastrointestinal tract, and they usually harbour an activating mutation in either *KIT* (about 75%) or the *platelet-derived growth factor alpha* (*PDGFRA*) (about 15%) gene [2].

GIST may rupture either spontaneously or at surgery spilling their contents into the abdominal cavity. GIST rupture is a serious prognostic feature, since the seeded cancer cells often give rise to intra-abdominal implant metastases, and the great majority of ruptured GISTs recur despite macroscopically complete surgery [3–6]. Recurrence after rupture is so frequent that there is uncertainty whether ruptured GISTs should be considered already metastatic cancers [7, 8]. Ruptured GISTs also frequently have other established adverse prognostic features, such as a high mitotic count, large size, and a non-gastric site of origin [1, 6]. In the modified National Institutes of Health (NIH) risk stratification scheme all ruptured GISTs are considered high-risk tumours [9]. About 5% of all GISTs and 12% of high-risk GISTs have ruptured [10].

Patients with ruptured GIST are recommended to be treated with adjuvant imatinib after surgery. The European Society for Medical Oncology (ESMO) guidelines recommend adjuvant imatinib for a duration of three years after surgery to patients with high-risk GIST [7], and the National Comprehensive Cancer Network of the U.S. guidelines at least for 3 years [11], but the optimal duration is unknown [7]. Some data from cohort studies suggest that patients with ruptured GIST may benefit from longer than 3 years of adjuvant imatinib [12–14], and even life-long imatinib has been suggested [7, 8].

The recommendation to administer adjuvant imatinib for 3 years to high-risk patients is largely based on the Scandinavian Sarcoma Group (SSG) XVIII/Arbeitsgemeinschaft Internistische Onkologie (AIO) trial, where patients with high-risk GIST were randomly allocated to receive adjuvant imatinib either for 1 year or 3 years after surgery [15–17]. In the latest analysis of the SSG XVIII/AIO trial the risk of death was 45% smaller in the 3-year group than in the 1-year group during a median of 10 years of follow-up after the date of randomisation, indicating a substantial overall survival (OS) benefit from the longer duration of adjuvant imatinib [16]. In the subset of patients with *KIT* exon 11 deletion/indel mutation, which are the most common mutations in GIST and considered imatinib-sensitive [18], 3-year adjuvant imatinib led to 66% reduction in the risk of death compared to 1 year of adjuvant imatinib [17]. The SSG XVIII/AIO trial also accrued patients whose disease is now considered insensitive to imatinib due to the absence of a *KIT* or *PDGFRA* mutation or the presence of imatinib-insensitive *PDGFRA* D842V mutation [7, 19].

To our knowledge, there is no information available from randomised trials about how adjuvant imatinib influences recurrence-free survival (RFS) or OS of patients with ruptured GIST. We investigated this and the impact of *KIT* mutational status on survival in the SSG XVIII/AIO trial patient population. We also reviewed the types of ruptures reported to the SSG XVIII/AIO trial database retrospectively, since GIST ruptures may range from minor defects to full-blown major ruptures, and since rupture classifications have now become available [8, 20].

## Patients and methods

### Study design and conduction

The SSG XVIII/AIO trial (NCT00116935) is a randomised, multicentre, open-label Phase 3 trial, where the participating patients were randomly allocated after surgery to receive adjuvant imatinib orally 400 mg daily either for 12 months or 36 months [15]. Based on the study power calculations a total of 400 patients were enroled, of whom 200 were assigned to the 1-year group and 200 to the 3-year group between February 4, 2004, and September 29, 2008 [15].

### Patient eligibility

The study participants were required to be ≥ 18 years of age, have the Eastern Cooperative Oncology Group performance status ≤ 2, and have undergone macroscopically complete resection of GIST at open surgery [15]. GIST was required to be KIT-positive at immunohistochemical evaluation. Patients who had received neoadjuvant therapy or had recurrent or metastatic disease were not eligible. Patients with completely excised intra-abdominal metastases were eligible until October 2006, but after the study protocol amendment, such patients were subsequently excluded.

The estimated risk of GIST recurrence was required to be high with one or more of the following criteria met: (1) diameter > 10 cm, (2) > 10 mitoses per 50 high power fields (HPFs), (3) diameter > 5 cm and the mitotic count > 5/50 HPFs, or (4) presence of GIST rupture [9].

### Study procedures

Randomisation was central, and the patients were allocated into 2 strata, either local disease (no tumour rupture and complete surgical tumour removal) or intra-abdominal disease (tumour rupture or R1 surgery with suspected microscopic residual tumour infiltration) [15]. Staging examinations included contrast-enhanced computed tomography (CT) or magnetic resonance imaging (MRI) of the abdomen and the pelvis, and chest CT or X-ray. The patients were scheduled for follow-up visits for up to 10 years since the date of randomisation [15]. The abdomen and the pelvis were imaged with CT or MRI 6-monthly during the first 7 years of follow-up, and then annually. The present analysis is based on the maximum follow-up obtainable in the trial, which was achieved when the last patient entered the trial had been followed up for 10 years [16].

GIST histology was reviewed centrally during the study by expert sarcoma pathologists. At the central review, 15 tumours were found not to be GISTs but usually another type of sarcoma [15]. *KIT* (HGNC:6342) exons 9, 11, 13, and 17, and *PDGFRA* (HGNC:8803) exons 12 and 18 were sequenced centrally using Sanger sequencing during the study [15].

### Tumour rupture classification

Since information about the type of GIST rupture was not captured into the SSG XVIII/AIO trial database, tumour ruptures were classified retrospectively after reviewing the medical case records as either a minor rupture or a major rupture according to the Oslo criteria [20] with minor modifications (supplementary Table S1). Tumour content spillage, tumour fracture, piecemeal resection, presence of blood-tinged ascites, microscopic infiltration of an adjacent organ, and a surgical biopsy were considered major ruptures, whereas bowel perforation without spillage into the peritoneal cavity was not considered a rupture. Peritoneal tumour penetration, iatrogenic peritoneal laceration, and a microscopically involved resection margin were considered minor ruptures [20].

### Statistical analysis

The primary endpoint in the trial was RFS, and OS was a secondary objective. RFS was defined as the interval between the date of randomisation and the date of GIST recurrence or death, whichever occurred first, patients alive with no recurrence were censored on the date of the last follow-up. OS was defined as the interval between the date of randomisation and the date of death, patients alive were censored. Survival was estimated with the Kaplan-Meier method, and survival between groups was compared with the log-rank test. The hazard ratios (HRs) and their confidence intervals (CI) were calculated with a univariable Cox model. The prognostic importance of GIST rupture compared with the three other factors included in the modified NIH risk assessment scheme (tumour size, mitotic count, and tumour site in the gastrointestinal tract) and the treatment group was analysed using Cox’s proportional hazard model. Frequency tables were analysed using the chi-square test, and continuous distributions were compared with the Mann-Whitney test. The *p* values are 2-sided and unadjusted for multiple testing. The statistical analyses were carried out with the IBM SPSS Statistics version 29 for windows.

## Results

### Study patient population and tumour rupture classification

**Fig. 1:**
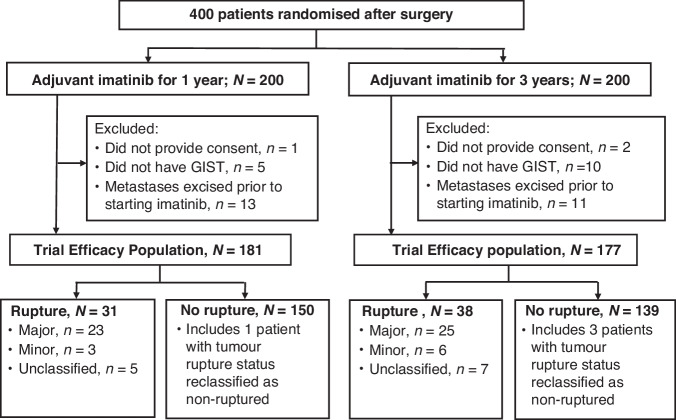
CONSORT diagram of the study population.

Of the 400 patients randomised, we excluded three patients who were randomised without signing informed consent, 15 patients who did not have GIST at the central review of tumour histology, and 24 patients who had intra-abdominal metastases resected at surgery, which left 358 patients in the trial Efficacy Population, 181 in the 1-year arm and 177 in the 3-year arm (Fig. 1). The median duration of imatinib treatment in the 1-year and the 3-year groups was 12.0 months and 36.0 months, respectively. None of the patients received adjuvant imatinib longer than 37.2 months.

Seventy-three (20.4%) of the 358 patients were reported to have GIST rupture to the trial database. In 12 (16.4%) of the 73 cases the medical records were not obtained for review. Forty-eight (78.7%) of the remaining 61 GISTs were classified as having undergone a major rupture, nine (14.8%) a minor rupture, and in four (6.6%) cases the tumour had ruptured into the bowel lumen without a spillage into the peritoneal cavity. These four GISTs were considered unruptured [8, 20] and were analysed together with 285 patients with non-ruptured GIST in the statistical analyses. Therefore, the final subgroup of patients with tumour rupture consisted of 69 patients, and the subgroup without a tumour rupture of 289 patients (Fig. 1). Most (*n* = 39, 56.5%) of the 69 ruptures occurred prior to surgery.

### Patient and tumour characteristics

The median age of the 358 patients was 61 years (range, 22 to 84 years), and 184 (51.4%) were male. Ruptured GISTs were more frequently non-gastric compared with non-ruptured tumours, and they harboured more frequently *KIT* exon 9 mutations (Table 1). There was no statistical difference in the distributions of tumour size or mitotic counts between the rupture group and the non-rupture group.

**Table 1:** Characteristics of the patients with and without tumour rupture.

Variable	No rupture *n* = 289	Rupture *n* = 69	*p*
Age - median (range)	61 (22–84)	58 (26–80)	0.406
Gender – No. (%)^a^			
Female	135 (78)	39 (22)	
Male	154 (84)	30 (16)	0.143
Primary tumour site - No. (%)			
Gastric	168 (88)	23 (12)	
Non-gastric	119 (72)	46 (28)	<0.001
Not available	2	0	
Primary tumour diameter - cm			
Median (range)	10 (2–40)	10 (2–22)	0.670
Not available	2	0	
Primary tumour mitotic count - No.^b^			
Median (range)	6 (0–135)	6 (0–54)	0.404
Not available	13	1	
Tumour mutation type – No.			
* KIT* exon 9	13 (50)	13 (50)	<0.001^c^
* KIT* exon 11	201 (82)	43 (18)	
*KIT* exon 11 del or indel	118 (79)	31 (21)	
*KIT* exon 11 substitution	60 (88)	8 (12)	
*KIT* exon 11 duplication/insertion	18 (82)	4 (18)	
* PDGFRA*	37 (86)	6 (14)	
*PDGFRA* exon 18 mutation D842V	27 (90)	3 (10)	
Other mutation	4 (100)	0 (0)	
Wild type for *KIT* and *PDGFRA*	19 (79)	5 (21)	
Not available	15	2	

*PDGFRA* platelet-derived growth factor receptor alpha gene.

^a^Percentages may not sum up to 100 due to rounding.

^b^Mitotic count was assessed centrally by one of two pathologists from 50 high-power fields. The total area of the 50 HPFs was either 11.24 mm^2^ or 12.50 mm^2^.

^c^The *p* value refers to the comparison of the frequency of *KIT* exon 9, *KIT* exon 11, and *PDGFRA* mutations between the non-ruptured and ruptured tumours.

Thirty-one (44.9%) of the 69 patients with rupture were allocated to adjuvant imatinib for 1 year and 38 (55.1%) for 3 years. The characteristics of the 69 patients and their tumours by the random allocation group are provided in Supplementary Table S2. Eighteen (26.1%) of the 69 patients with rupture discontinued adjuvant imatinib before the scheduled duration was reached. Eight (44.4%) of these 18 patients stopped imatinib since GIST recurred while the patient was on imatinib (all in the 3-year group). One patient in the 1-year group stopped taking imatinib after 2.6 months, and in the 3-year group the median duration of imatinib administration was 21.0 months (range, 3.7–33.7 months) in the subset of nine patients who stopped imatinib for another reason than GIST progression.

### Survival of patients with GIST rupture

**Fig. 2:**
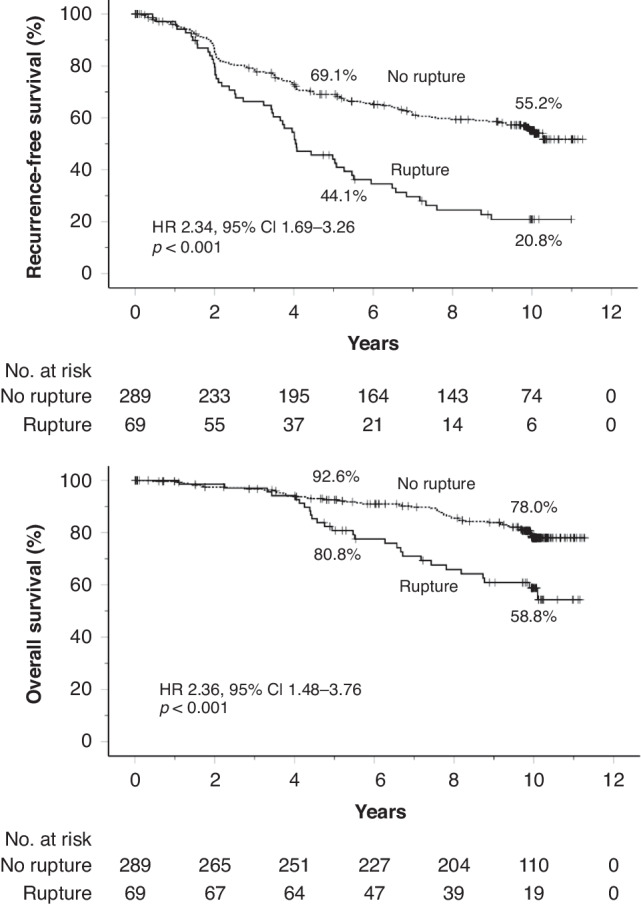
Survival outcomes of patients with and without tumour rupture. Upper panel: recurrence-free survival; lower panel: overall survival. Five-year and 10-year survival rates are shown. Patients alive are indicated with a bar.

Fifty-two (75.4%) RFS events occurred in the subset of 69 patients with rupture and 119 (41.2%) among the 289 patients with no rupture during a median follow-up time of 10.0 years, and 27 (39.1%) and 53 (18.3%) patients died, respectively. Patients with rupture had inferior RFS and OS compared with patients with non-ruptured GIST (HR 2.34, 95% CI, 1.69–3.26; *p* < 0.001; and HR 2.36, 95% CI, 1.48–3.76; *p* < 0.001, respectively; Fig. 2). Of the patients with rupture, 20.8% were alive without recurrence 10 years after the date of randomisation, and 58.8% were alive. The RFS and OS of the patients with minor rupture were also inferior compared to patients with no rupture (HR 2.24, 95% CI, 1.04–4.81; *p* = 0.039, and HR 2.82, 95% CI, 1.02–7.81; *p* = 0.037, respectively). The RFS and OS of the 12 patients whose rupture could not be classified resembled those of the patients with minor rupture (supplementary Fig. S1). In a multivariable analysis that contained tumour rupture (rupture vs. no rupture), tumour site (non-gastric vs. gastric), mitotic count (continuous covariable), size (continuous covariable) and the treatment group (1-year vs. 3-years) as covariables, presence of GIST rupture was independently associated with unfavourable RFS (HR 2.34, 95% CI 1.66–3.28; *p* < 0.001) together with non-gastric tumour site (HR 2.91, 95% CI 2.10–4.07; *p* < 0.001), large GIST size (HR 1.05, 95% CI 1.03–1.08; *p* < 0.001), a high mitotic count (HR 1.03, 95% CI 1.02–1.03; *p* < 0.001), and the 1-year treatment group (HR 1.61, 95% CI 1.17–2.20; *p* = 0.003). GIST rupture was independently associated also with unfavourable OS (HR 2.50, 95% CI 1.53–4.08; *p* < 0.001) as were the mitotic count (HR 1.02, 95% CI 1.01–1.03; *p* < 0.001), the 1-year treatment group (HR 1.98, 95% CI 1.22–3.20; *p* = 0.006), and a non-gastric tumour site (HR 1.68, 95% CI 1.05–2.70; *p* = 0.032), whereas tumour size was not (*p* = 0.301). When age at study entry was added as the sixth covariable to this analysis, GIST rupture was still independently associated with OS (Supplementary Table S3).

### Influence of adjuvant imatinib on survival

**Fig. 3:**
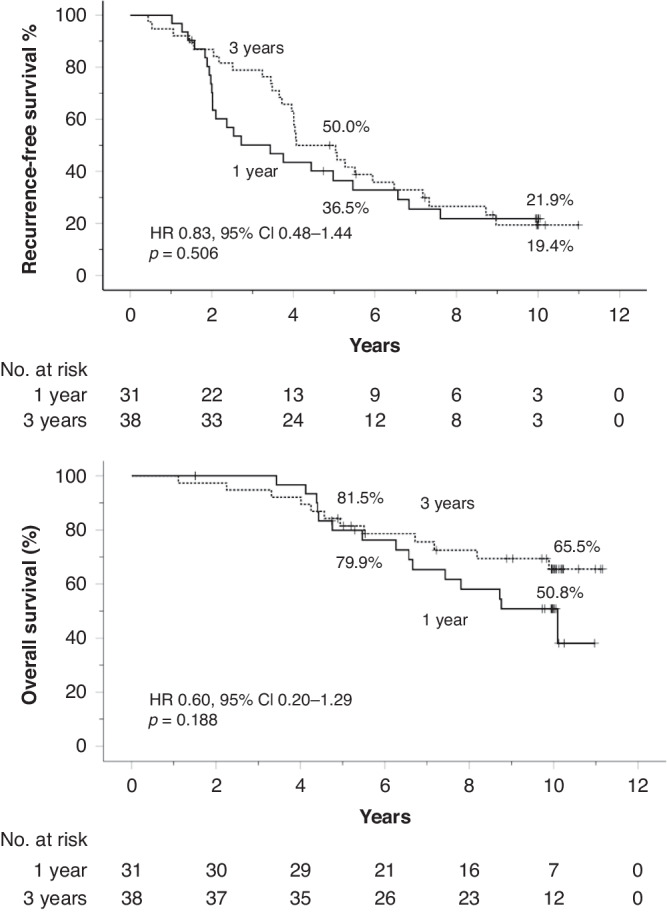
Influence of the duration of adjuvant imatinib on survival outcomes of patients with tumour rupture. Upper panel: recurrence-free survival; lower panel: overall survival. Five-year and 10-year survival rates are shown. Patients alive are indicated with a bar.

There was no significant difference in RFS or OS in the subset of patients with ruptured GIST when the patients assigned to 3-year adjuvant imatinib were compared to those assigned to 1-year of imatinib (Fig. 3). The results remained essentially similar when these analyses were restricted to patients with major rupture (Supplementary Fig. S2).

### *KIT* exon 11 deletion/indel mutations and survival

**Fig. 4:**
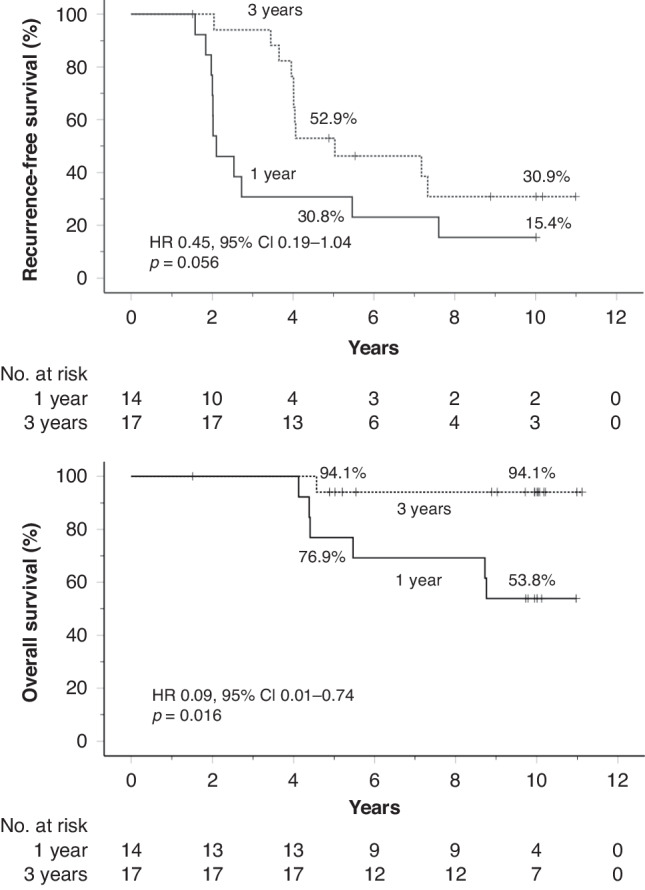
Influence of the duration of adjuvant imatinib on survival outcomes of patients with *KIT* exon 11 deletion/indel mutation and with tumour rupture. Upper panel: recurrence-free survival; lower panel: overall survival. Five-year and 10-year survival rates are shown. Patients alive are indicated with a bar.

We next investigated the survival of patients with ruptured GIST in the largest mutational subgroup, patients with *KIT* exon 11 deletion/indel mutation. Thirty-one (46.3%) of the 67 patients with rupture and with mutation analysis results available had *KIT* exon 11 deletion/indel mutation. Patients allocated to 3 years of imatinib tended to have longer RFS compared to those allocated to 1 year of imatinib in this mutational subgroup (HR 0.45, 95% CI, 0.19–1.04; *p* = 0.056). A large drop in RFS occurred in the Kaplan-Meier plots once imatinib was stopped (Fig. 4). The patients allocated to 3-year adjuvant imatinib had longer OS than those allocated to 1 year of adjuvant imatinib when GIST harboured *KIT* exon 11 deletion/indel mutation (HR 0.09, 95% CI, 0.01–0.74; *p* = 0.016). Only 1 of the 17 patients assigned to the 3-year adjuvant imatinib group died during the follow-up despite a ruptured tumour, which resulted in a high 10-year OS rate of 94.1% (90.0% when the analysis was restricted to patients with a major rupture; supplementary Fig. S3).

The numbers of patients with some other type of *KIT* or *PDGFRA* mutation or no mutation in these genes were too small for carrying out reliable survival analyses. All 13 ruptured GISTs with *KIT* exon 9 mutation recurred.

### Treatment after GIST recurrence

The treatments for recurred GIST were administered after the trial primary endpoint had been met and outside of the trial protocol. Despite the treatments for recurred or overtly metastatic GIST were selected based on the institutional practice, we collected limited data about them when feasible, since they likely influence OS. Information about the first-line systemic treatment after GIST recurrence was available from 47 (68.1%) of the 69 patients with rupture. Thirty-four (72.3%) of the 47 patients received imatinib as the first-line treatment for advanced GIST, four (8.5%) sunitinib, three (6.4%) nilotinib, and six (12.8%) patients received no systemic treatment. Therefore, a total of 41 (87.2%) of the 47 patients with information available received tyrosine kinase inhibitor treatment after GIST recurrence as their first-line treatment for recurrent GIST.

## Discussion

Patients with ruptured GIST had inferior RFS and OS compared with other high-risk patients who participated in the SSG XVIII/AIO trial. While ruptured GISTs recur almost invariably after surgery alone [3, 4], about 20% of the patients with any rupture and about 20% of those with confirmed major rupture did not have GIST recurrence during a median follow-up of 10 years when adjuvant imatinib was administered after surgery. The great majority of GIST recurrences occur within the first 10 years that follow surgery [4]. Only one of the 17 patients with *KIT* exon 11 deletion/indel mutation and assigned to the 3-year adjuvant imatinib group died leading to a 94% 10-year OS rate in this subset of patients. To our knowledge, this is the highest 10-year OS rate reported in a patient subpopulation with ruptured GIST.

Several factors may have contributed to the relatively high OS rate observed in the subset with *KIT* exon 11 deletion/indel mutation. GISTs with *KIT* exon 11 deletion/indel mutation usually respond to imatinib and are considered generally imatinib-sensitive [18]. Most patients with ruptured GIST were treated after recurrence with tyrosine kinase inhibitors that are known to be effective for advanced GIST [7, 12]. The SSG XVIII/AIO trial participants were followed up longitudinally using CT or MRI imaging, which allowed detection of asymptomatic recurrent disease when the tumour burden was still small possibly lowering the risk of rapid emergence of drug-resistant tumour clones.

There is uncertainty of whether patients with ruptured GIST benefit from longer than three years of adjuvant imatinib [7]. We found no statistical difference in RFS or OS between the 1-year and 3-year treatment durations in the group of patients with ruptured GIST in a univariable analysis, but this analysis may have been underpowered. The treatment group was an independent prognostic factor for both RFS and OS in multivariable analyses that contained the prognostic factors considered most important in GIST [4]. Furthermore, there were large abrupt drops in the Kaplan-Meier plots for RFS observed in the subgroup of patients with *KIT* exon 11 deletion/indel mutation after stopping adjuvant imatinib suggesting that some patients could have benefitted from longer adjuvant treatment (Fig. 4). The results from a few cohort studies suggest that 5-year adjuvant imatinib might be superior to the 3-year duration in the treatment of patients with GIST rupture [12–14]. On the other hand, a high 10-year OS rate was achieved among patients with *KIT* exon 11 deletion/indel mutation with 3-year adjuvant imatinib and with the subsequent treatments administered after GIST recurrence. The two ongoing randomised trials (NCT02260505 and NCT02413736) comparing 3-year adjuvant imatinib with longer durations in patient populations with high-risk GIST may provide further guidance.

We classified the ruptures as minor or major since minor ruptures impact survival less than major ruptures [5]. We found that patients with a minor rupture had inferior RFS and OS compared to patients with no rupture. This finding needs to be interpreted cautiously, because only nine patients had a minor rupture increasing the risk of this observation arising by chance, and there could sometimes be difference of opinion in rupture classification. Nevertheless, the findings of the study were essentially similar in the entire cohort of 69 patients and in the subset of 48 patients with a major rupture. We classified four GISTs originally reported as ruptured tumours based on the presence of bowel lumen perforation as unruptured, since such perforations do not lead to peritoneal seeding and should not be considered ruptures [5, 8].

The study has a few limitations. The numbers of patients with ruptured GIST in the subsets were relatively small, particularly in the mutational subsets. This is a common limitation in studies on ruptured GIST due to the infrequency of these tumours. The ruptures were classified retrospectively, because the rupture classifications were developed only recently and the details required for rupture classification were not captured during the trial. The case records of 12 patients with ruptured GIST could not be obtained for review. The records of the patients with no reported rupture were not reviewed, which could have left some ruptures unidentified. We reached the maximum patient follow-up time attainable in the trial, but the median of 10-year follow-up time may still be short for assessing OS, because adjuvant imatinib may delay GIST recurrence. The strengths of the study include the randomised trial setting with monitored trial data, protocol-defined patient population and treatments, and central tumour histology review and mutation analysis.

In conclusion, patients with ruptured GIST have inferior RFS and OS compared to other high-risk patients when treated with adjuvant imatinib. About one fifth of the SSG XVIII/AIO trial patients with ruptured GIST did not have GIST recurrence during the first decade of follow-up. The optimal duration of adjuvant imatinib in the subset of patients with GIST rupture remains unknown. A high 10-year OS rate was achieved in the 3-year adjuvant imatinib group when GIST harboured an imatinib-sensitive *KIT* exon 11 deletion/indel mutation. Yet, studies that evaluate longer than the 3-year duration of adjuvant imatinib are needed also in this subgroup of patients.

### Supplementary information

Supplement

## Data Availability

The study protocol has been available since the time of the trial primary publication. Considering patients’ privacy and related regulations in the countries that participated in the SSG XVIII/AIO trial, we do not make the database public. Address requests for the database to the corresponding author. Reasonable requests will be evaluated and approved by the SSG XVIII/AIO trial Steering Committee.
