# Supplementary material for: Survival of patients with ruptured gastrointestinal stromal tumour treated with adjuvant imatinib in a randomised trial
Source: Br J Cancer. 2024 Jun 11;131(2):299–304. doi: 10.1038/s41416-024-02738-z (PMC11263706; doi:10.1038/s41416-024-02738-z)
Supplement: Supplementary file 1 — Supplement [file 41416_2024_2738_MOESM1_ESM.pdf]

Supplement

Supplementary Table S1. Modifications to the Oslo tumour rupture classification used in the study.

**Major defect**
• Spillage can occur either before surgery or at surgery.<br>• Tumour fracture: tumour split into two or more fragments.<br>• Piecemeal resection: the surgical resection line cuts the macroscopic tumour.
• Bowel perforation without spillage into the peritoneal cavity is not considered tumour rupture.
• Blood-tinged ascites, or blood in the abdominal cavity prior to starting tumour resection.
• Excisional biopsy, a part of the tumour was removed at surgery for a biopsy. A needle biopsy is not considered a surgical biopsy regardless of the needle diameter.
**Minor defect**
• Peritoneal tumour penetration the peritoneal membrane is involved, but there is no tumour growth into the adjacent organs.
• Iatrogenic peritoneal laceration may cause bleeding, but there is no macroscopic spillage of the tumour content into the abdominal cavity.
• Involved resection margin: R1 resection

Supplementary Table S2. Characteristics of patients with tumour rupture by the random allocation group**.**

**Variable**	**12-month group** $n = 31$	**36-months group** $n = 38$
Age - median (range)	61 (30-80)	57 (26-78)
Gender - No. (%)		
Female	17 (44)	22 (56)
Male	14 (47)	16 (53)
Primary tumour site - No. (%)		
Gastric	10 (43)	13 (57)
Non-gastric	21 (46)	25 (54)
Primary tumour diameter - cm		
Median (range)	8 (4-22)	11 (2-21)
Primary tumour mitotic count - No.[a]		
Median (range)	9 (0-54)	4 (0-48)
Not available	0	1
Tumour mutation type - No. (%)		
KIT exon 9	4 (31)	9 (69)
KIT exon 11	20 (47)	23 (53)
KIT exon 11 del or indel	14 (45)	17 (55)
KIT exon 11 substitution	3 (38)	5 (62)
KIT exon 11 duplication/insertion	3 (75)	1 (25)
PDGFRA	2 (33)	4 (67)
PDGFRA exon 18 mutation D842V	2 (67)	1 (33)
Wild type for *KIT* and *PDGFRA*	3 (60)	2 (40)
Not available	2	0
Type of rupture - No. (%)		
Major	23 (48)	25 (52)

Minor	3 (33)	6 (67)
Unclassified	5 (42)	7 (58)

Abbreviations: *PDGFRA*, platelet-derived growth factor receptor alpha gene.

[a]Mitotic count was assessed centrally by one of two pathologists from 50 high-power fields (HPFs). The total area of the 50 HPFs was either 11.24 mm^2 or 12.50 mm^2.

Supplementary Table S3. A Cox multivariable model for overall survival with six covariables**.**

**Covariable**	**HR (95% CI)**	***p***
Tumour site (nongastric vs. gastric)	2.08 (1.29-3.35)	0.003
Tumour size (cm, continuous)	1.01 (0.98-1.06)	0.491
Tumour mitotic count (counts, continuous)	1.02 (1.01-1.03)	<0.001
Tumour rupture (yes vs. no)	2.63 (1.62-4.28)	<0.001
Treatment group (1 year vs. 3 years)	1.92 (1.19-3.11)	0.008
Age at study entry (years, continuous)	1.04 (1.02-1.07)	<0.001

Abbreviations: HR, hazard ratio; CI, confidence interval.

Fig. S1

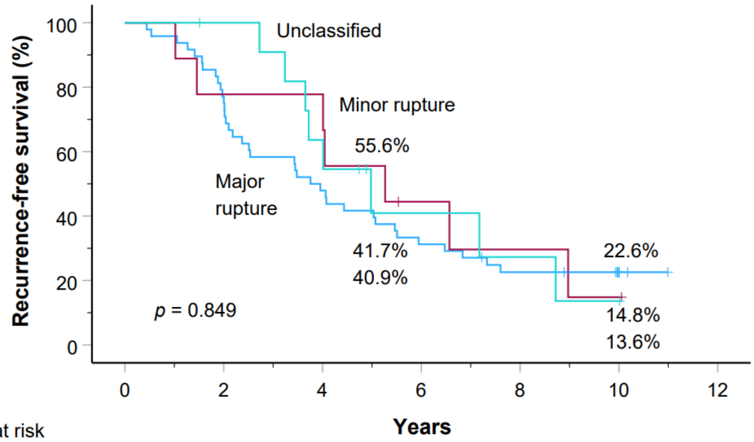

No. at risk							
Major	48	37	23	15	10	4	0
Minor	9	7	7	3	2	1	0
Unclassified	12	11	7	3	2	1	0

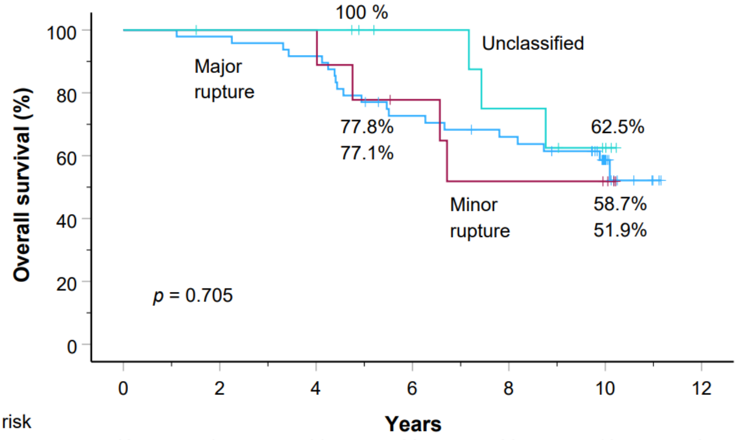

No. at risk							
Major	48	47	44	33	29	13	0
Minor	9	9	9	6	4	3	0
Unclassified	12	11	11	8	6	3	0

Figure S1. Survival outcomes of patients stratified by the type of tumour rupture. Upper panel: recurrence-free survival; lower panel: overall survival. Five-year and 10-year survival rates are shown. Patients alive are indicated with a bar.

Fig. S2

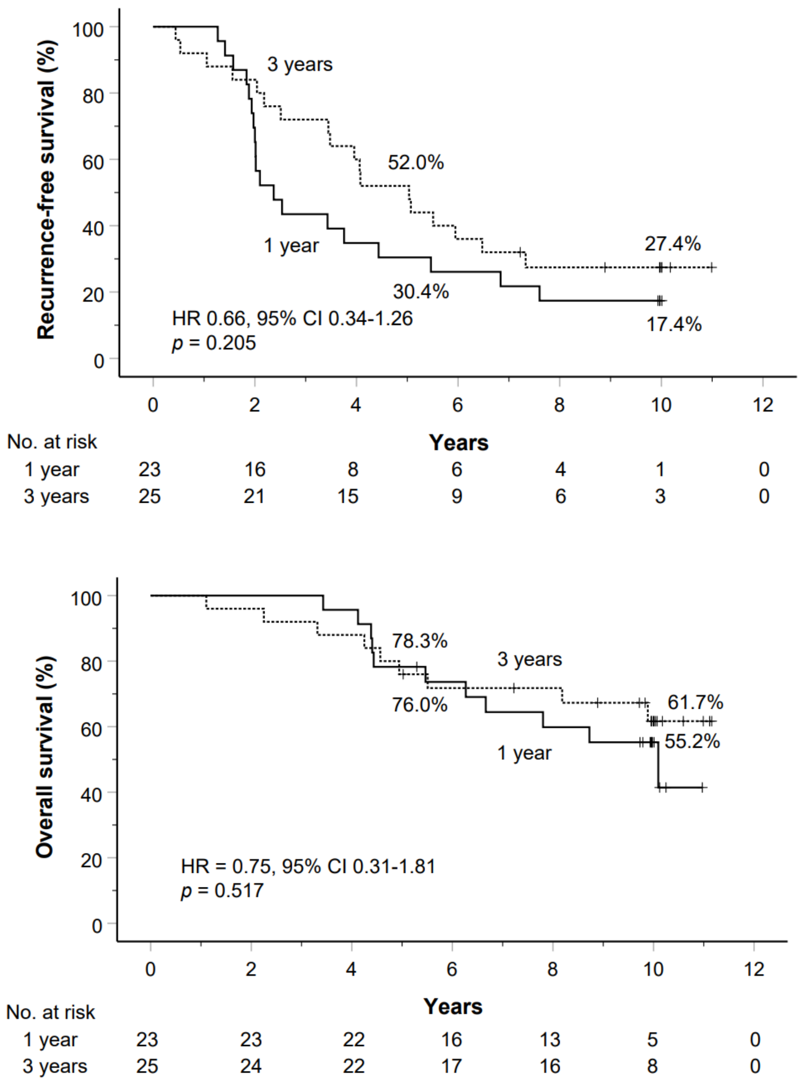

Figure S2. Influence of the duration of adjuvant imatinib on survival outcomes of patients with major tumour rupture. Upper panel: recurrence-free survival; lower panel: overall survival. Five-year and 10-year survival rates are shown. Patients alive are indicated with a bar.

Fig. S3.

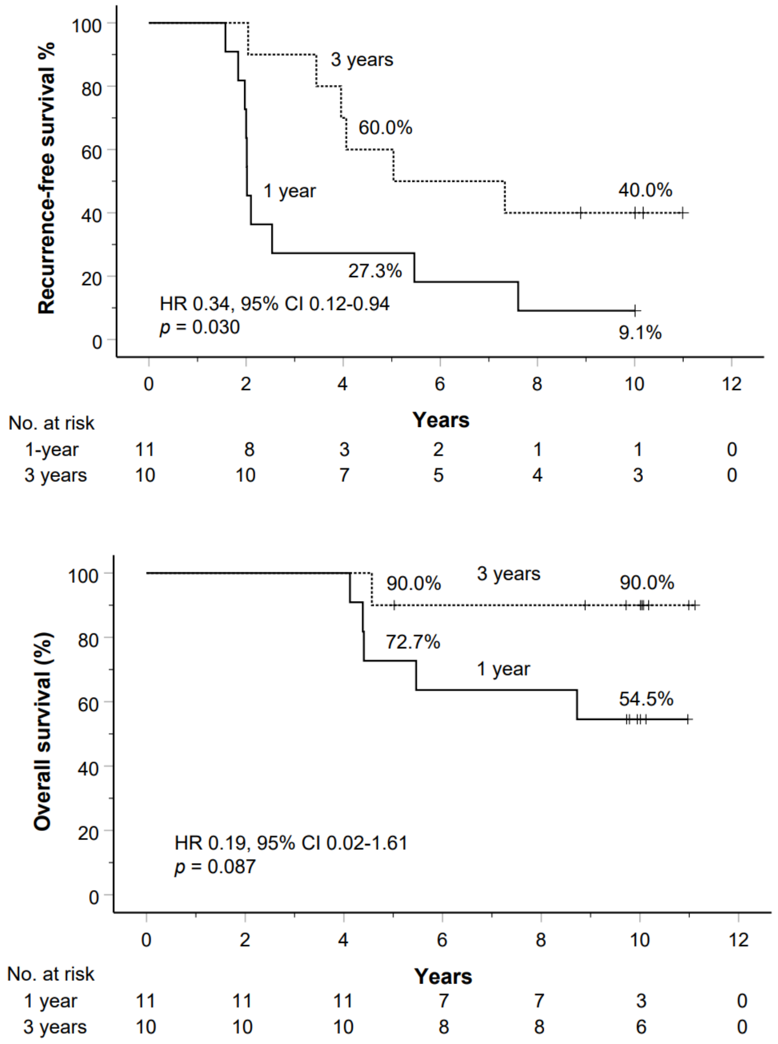

Figure S3. Influence of the duration of adjuvant imatinib on survival outcomes of patients with *KIT* exon 11 deletion/indel mutation and with major tumour rupture. Upper panel: recurrence-free survival; lower panel: overall survival. Five-year and 10-year survival rates are shown. Patients alive are indicated with a bar.
